# Supplementary material for: Search for Synergistic Drug Combinations to Treat Chronic Lymphocytic Leukemia
Source: Cells. 2022 Nov 18;11(22):3671. doi: 10.3390/cells11223671 (PMC9688317; doi:10.3390/cells11223671)
Supplement: Supplementary file 1 [file cells-11-03671-s001.zip › cells-1971371-supplementary.pdf]

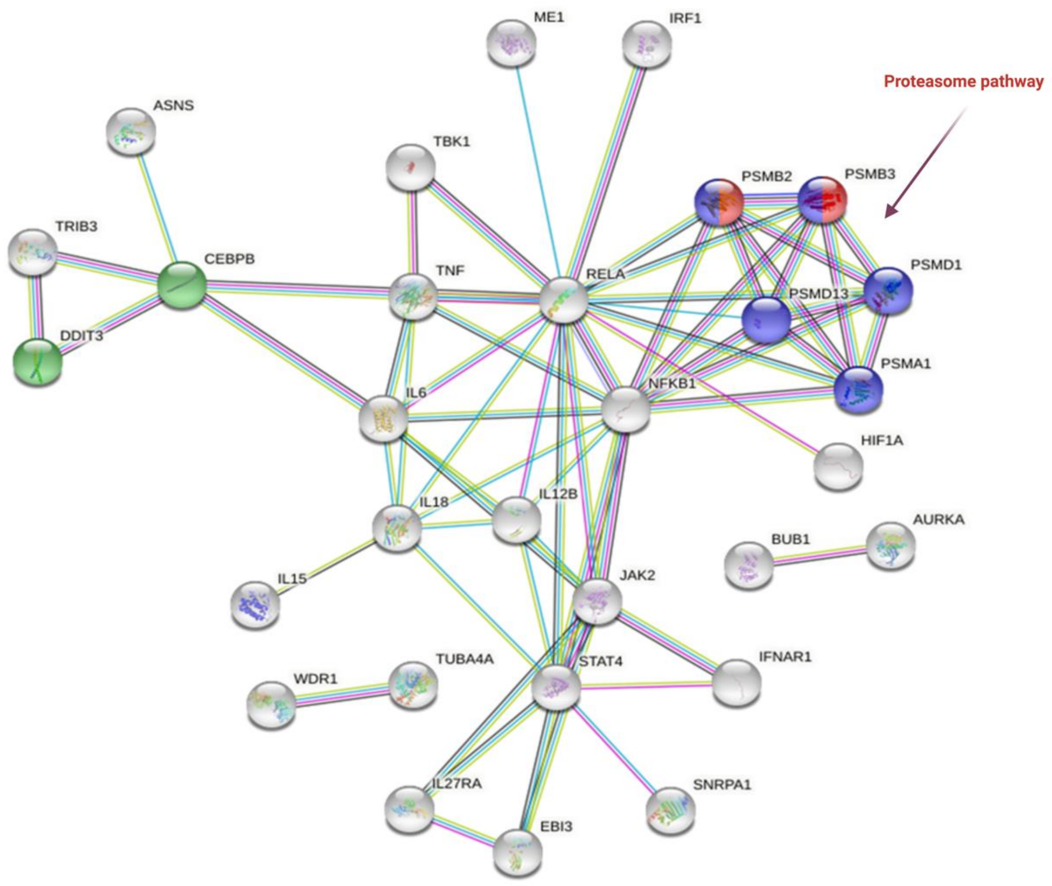

*GO-term	description	Count in network	Strength	False discovery rate
GO:0036488	CHOP-C/EBP complex	2/2	2.7	0.0027
GO:0033256	I-KappaB/NF-kappaB complex	2/7	2.16	0.0124
GO:0019774	Proteasome core complex, beta-subunit complex	2/11	1.96	0.0240
GO:0005839	Proteasome core complex	3/20	1.88	0.0027
GO:0000502	Proteasome complex	5/62	1.61	0.00018
GO:0000775	Chromosome centromere region	4/193	1.02	0.0456

* Cellular Component (Gene ontology).

Figure S1. STRING network analysis of genes that were involved in pathways identified by GSEA. The proteasome pathway is highlighted**.**

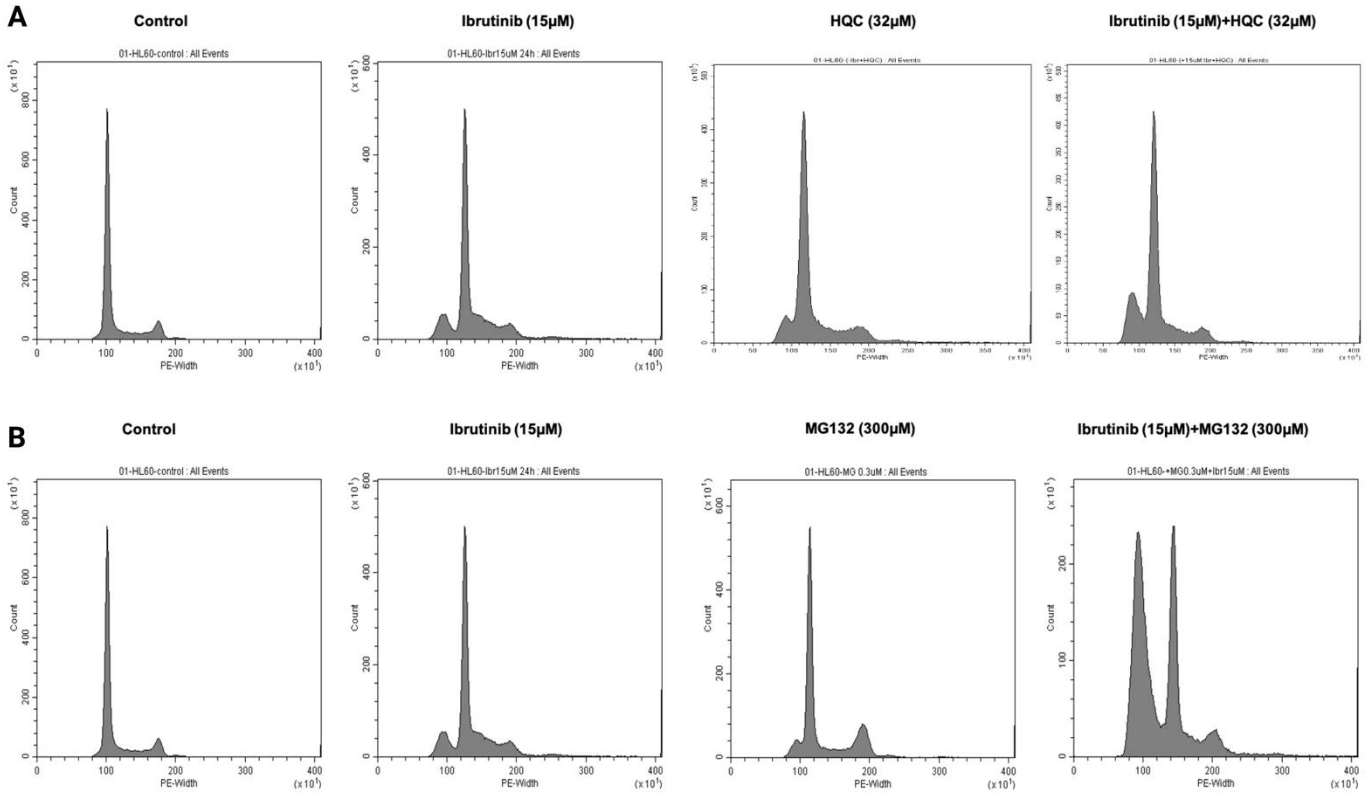

Figure S2. FACS quantification for Control (Untreated), Ibrutinib (15uM), and combination of Ibrutinib with HQC and MG132, raw data sub-G1 in experiments described in Fig. 1B for HL-60 cell line. Here for representation, the data for control and Ibrutinib has been used twice (The first 2 images in the up and down panel).

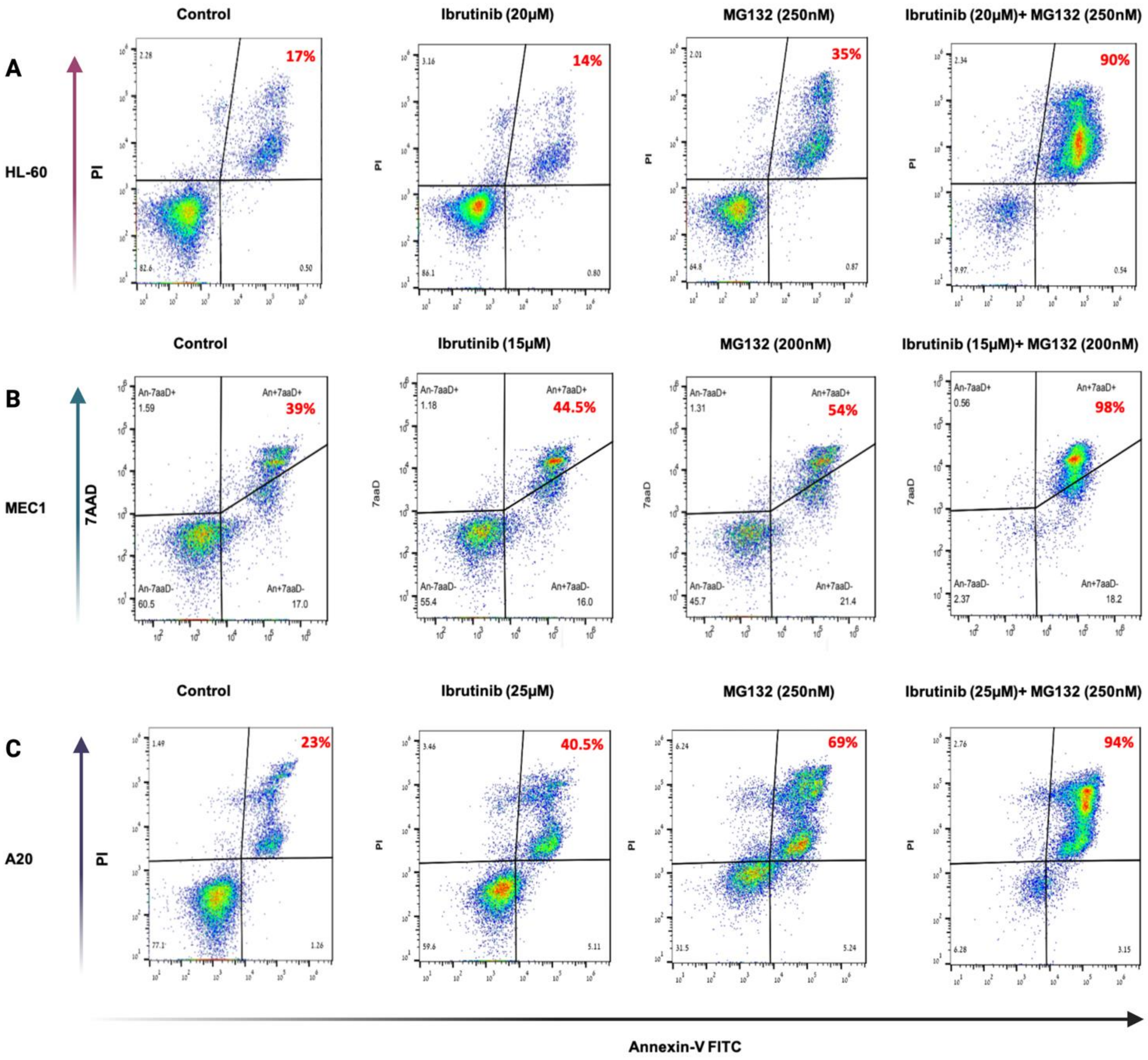

Figure S3. Raw data of the Annexin V tests described in Fig. 1C. (***A)*** Effect of ibrutinib and MG132 on HL-60 (***B)*** Effect of ibrutinib and MG132 on MEC1, (***C)*** Effect of ibrutinib and MG132 on A20.

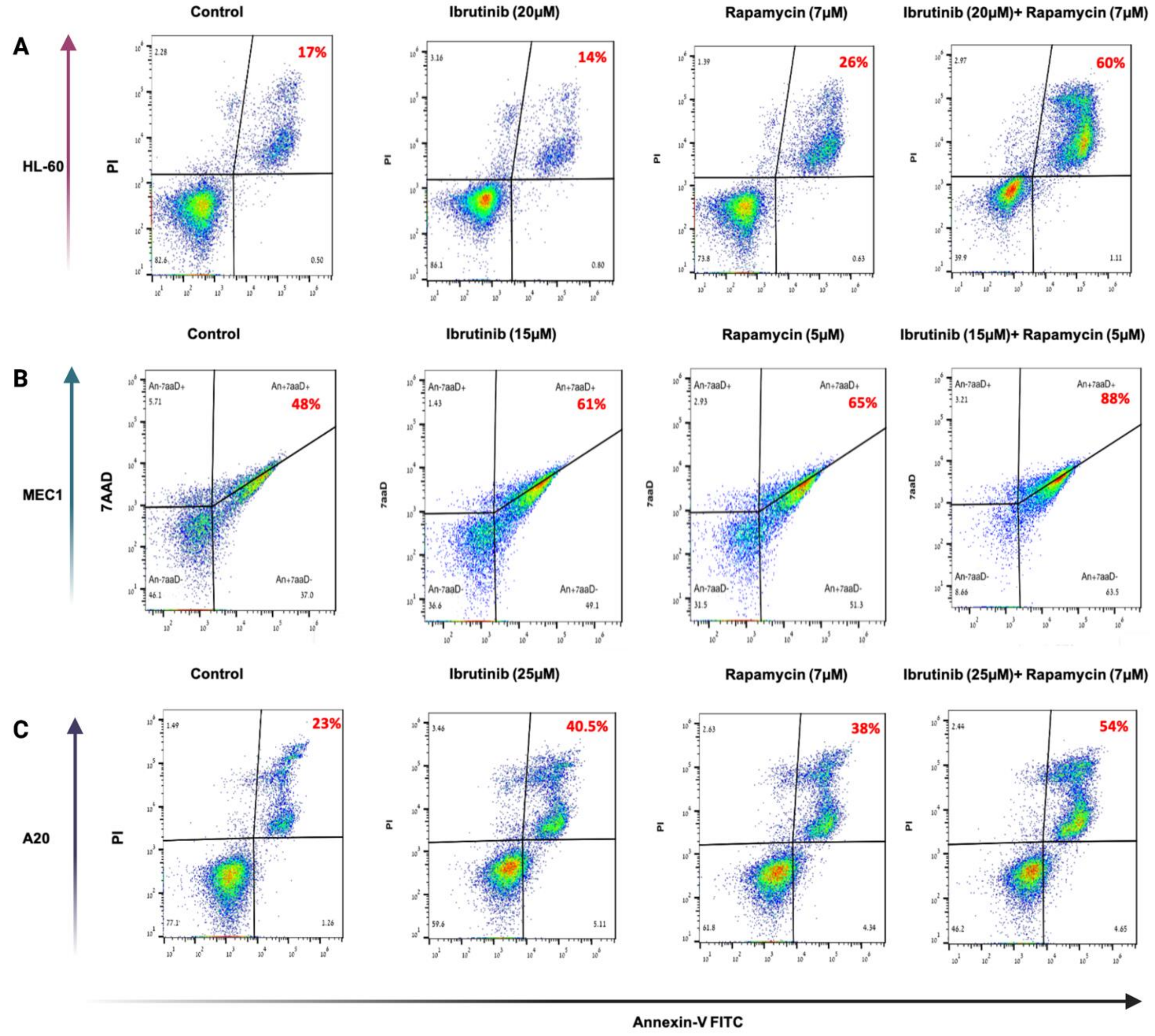

Figure S4. Raw data of the Annexin V tests described in Fig. 1C. (**A)** Effect of ibrutinib and Rapamycin on HL-60 (**B)** Effect of ibrutinib and Rapamycin on MEC1 **(C)** Effect of ibrutinib and Rapamycin on A20. Images for Controls in HL60 and A20 as well as Ibrutinib treatments are similar as described in Figure S4 as these are from the same experiments, we have used this data in this panel for representation and sidewise comparison.

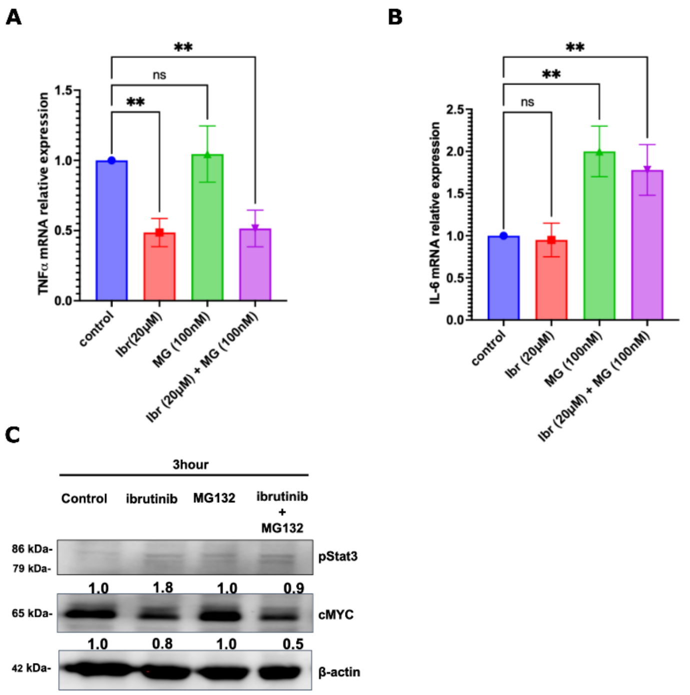

Figure S5. Relative normalized expression of **(A)** TNFα and **(B)** IL-6 mRNA in response to ibrutinib and MG132 was quantified through qPCR. The experiment was performed in biological triplicates (n=3), Statistical analysis for (A) and (B) was performed using one-way ANOVA. All statistical analysis was performed using Graphpad (v9), level of significance was taken as ("ns" < .1234, **"*P*" < .0021) as denoted appropriately on the above graphs. **(C)** MEC1 cells were treated with ibrutinib and MG132 or in combination, Levels of c-Myc and phopho-Stat3 were assessed by immunoblotting with antibodies, and Actin serves as a normalizing control.

Table S1. and S2. Sequences of primers used for amplifying the shRNA barcodes (***Table S1***) and for relevant genes expression (***Table S2***).

Table S1. Sequences of first and second PCR primers.

Primers	Sequence
1st PCR Forward	5′-TTCTCTGGCAAGCAAAAGACGGCATA-3′
1st PCR Reverse	5′-TAGCCAACGCATCGCACAAGCCA -3′
2nd PCR Reverse	5′-CCACTACGCCTCCGCTTTCCTCTCTATGGGCAGTCGGTGATAATGATACGGCGACCAC-CGAGA-3′
2nd PCR Forward Barcode 16	5′-CCATCTCATCCCTGCGTGTCTCCGACTCAGGTACGAGATCAAGCAGAAGACGGCAT-ACGAGA-3′
2nd PCR Forward Barcode 17	5′-CCATCTCATCCCTGCGTGTCTCCGACTCAGGTCGTAGATCAAGCAGAAGACGGCAT-ACGAGA-3′
2nd PCR Forward Barcode 18	5′-CCATCTCATCCCTGCGTGTCTCCGACTCAGGCTCACGATCAAGCAGAAGACGGCAT-ACGAGA-3′
2nd PCR Forward Barcode 19	5′-CCATCTCATCCCTGCGTGTCTCCGACTCAGCCATAGGATCAAGCAGAAGACGGCAT-ACGAGA-3′

Table S2. RT-PCR primers.

Primers for	Forward sequence	Reverse sequence
CHOP mouse	5′-CTGCCTTTCACCTTGGAGAC-3′	5′-CGTTTCCTGGGGATGA-GATA-3′
ATF4 mouse	5′-CCTGAACAGCGAAGTGTTGG-3′	5′-TGGAGAACCCATGAGGTTTCAA-3′
ATF3 mouse	5′-TTTGCTAACCTGACACCCTTTG-3′	5′-AGAGGACATCCGATGGCAGA-3′
HPRT-R mouse	5′-TGTTGTTGGATATGCCCTTG-3′	5′-TTGCTCATCTTAGGCTTT-3′
IL-6 human	5′-ACTCACCTCTTCAGAACGAATTG-3′	5′-CCATCTTTGGAAGGTTCAGGTTG-3′
TNFα human	5′-GATTCTGAGCAAAATAGCCAGCA-3′	5′-GGCTTCCTTCTTGTTGTGTGT-3′
Actin human	5′-CATGTACGTTGCTATCCAGGC-3′	5′-CTCCTTAATGTCACGCACGAT-3′

Table S3. A list of sensitized genes from statistical data is attached to an excel file (***Sensitizers_genetic_screen.xlsx***).

Table S4. List of significant pathways identified in the screen.

NAME	FDR q-val
HALLMARK_MYC_TARGETS_V1	0
HALLMARK_COAGULATION	0
HALLMARK_OXIDATIVE_PHOSPHORYLATION	0
HALLMARK_COMPLEMENT	0
HALLMARK_APOPTOSIS	0
HALLMARK_ALLOGRAFT_REJECTION	7.58E-04
HALLMARK_MTORC1_SIGNALING	0.0010850341
HALLMARK_DNA_REPAIR	0.0011429532
HALLMARK_FATTY_ACID_METABOLISM	0.0036229694
HALLMARK_PI3K_AKT_MTOR_SIGNALING	0.0038951442
HALLMARK_INFLAMMATORY_RESPONSE	0.0036818024
HALLMARK_IL6_JAK_STAT3_SIGNALING	0.0039023084
HALLMARK_PEROXISOME	0.021172734
HALLMARK_GLYCOLYSIS	0.019761236
HALLMARK_CHOLESTEROL_HOMEOSTASIS	0.025605407

Table S5. List of nine FDA approved drugs that were tested for synergy with ibrutinib, related inhibition pathways and genes from the screen.

Drug	The inhibition Pathway	Genes from our screen involved in the pathway
Hydroxychloroquine (HQC)	Autophagy	TNF, IL6, TNFAIP3, TRAF1, TRAF4, NFKB1, NFKB2
Tofacitinib	JAK-STAT	JAK2, STAT3, STAT2, GPR1
SP600125	JNK	MAPK8IP1, TNF, IL6
SB239063	p38 MAPK	NFKB1, NFAT
Fx 9847	DYRK	PTEN, CYCS, DYRK1B
Ex 527	SIRT1	SIRT1, SIRT2, TP53, CDK2, PLAG2A
17-AAG	HSP90	HSP90AB1, CASP3, HDAC9, PARP1, PARP2
MG132	Ubiquitin-proteasome	PSMA1, PSMA6, PSMA7, PSMD7, PSMD8, PSMB2, PSMB3
Rapamycin	mTORC1	RPTOR, RPS6KA1, EIF4E

Table S6. Synergy results of sub-toxic concentrations of the drugs with ibrutinib.

Drug	Sub-toxic concentration	Synergy with 15μM ibrutinib
Hydroxychloroquine (HQC)	30μM	+
Tofacitinib	10μM	-
SP600125	10μM	-
SB239063	10μM	-
Fx9847	2μM	-
Ex527	10μM	-
17-AAG	100nM	-
MG132	100nM<br>300nM	+<br>+++
Rapamycin	5-10μM	++

Table S7. Combination Index.

Cell line	IC50	Combination Index
HL-60	Ibrutinib 20μM+MG132 150nM	0.95
MEC1	Ibrutinib 15μM+MG132 150nM	0.86
HL-60	Ibrutinib 20μM+Rapamycin 5μM	0.91
MEC1	Ibrutinib 15μM+Rapamycin 5μM	0.93

Table S8. Up-regulated pathways in response to ibrutinib in HL-60 cells.

UPREGULATED PATHWAYS	FDR
TNFa SIGNALING VIA NFKB	0.000
P53 PATHWAY	0.000
HYPOXIA	0.000
APOPTOSIS	0.001
EPITHELIAL MESENCHYMAL TRANSITION	0.006
MTORC1 SIGNALING	0.007
DNA REPAIR	0.007
IL6 JAK STAT3 SIGNALING	0.039
GLYCOLYSIS	0.099
PI3K AKT MTOR SIGNALING	0.126
UNFOLDED PROTEIN RESPONSE	0.127
TGF BETA SIGNALING	0.136
KRAS SIGNALING	0.163
UV RESPONSE UP	0.185
XENOBIOTIC METABOLISM	0.187
FATTY ACID METABOLISM	0.196
IL2 STAT5 SIGNALING	0.250

Table S9. Down-regulated pathways in response to ibrutinib in HL-60 cells.

DOWNREGULATED PATHWAYS	FDR
HALLMARK_OXIDATIVE_PHOSPHORYLATION	0.092
HALLMARK_E2F_TARGETS	0.126
